# Psychological factors of adherence to treatment in patients with cardiovascular diseases

**DOI:** 10.1192/j.eurpsy.2025.1229

**Published:** 2025-08-26

**Authors:** E. V. Deshchenko, E. I. Pervichko, E. V. Akatova, O. P. Nikolin

**Affiliations:** 1 Lomonosov Moscow State University; 2 Russian University of Medicine of the Ministry of Health of Russia, Moscow, Russian Federation

## Abstract

**Introduction:**

Cardiovascular diseases are one of the leading causes of death worldwide. Adherence to treatment is considered to be a key determinant of the effectiveness of therapy. Patient’s personality and other psychological characteristics play a regulating role in health behaviour but there is still no consistent evidence on their connection to the adherence to treatment.

**Objectives:**

The study aimed to determine the role of psychological factors regarding the adherence to treatment in patients with cardiovascular diseases.

**Methods:**

Adherence was measured using the Questionnaire for Comprehensive Assessment of Treatment Adherence (Nikolayev *et al*. Clinical Pharmacology and Therapy 2018, 1 74-78). To provide a complex assessment of psychological factors we used the Short Health Anxiety Inventory (Salkovskis *et al*. Psychological Medicine 2002, 32 843-853; Pervichko, Shishkova. National Psychological Journal 2022, 2), the HEXACO Personality Inventory (Ashton, Lee. Personality and Social Psychology Review 2007, 11 150-166; Egorova *et al*. Issues of Psychology 2019, 5 33-49), the Defense Mechanisms Rating Scales (DMRS-SR-30; Di Giuseppe *et al*. Front. Psychiatry 2020, 11:870), and the Picture Frustration Test (Rosenzweig. Journal of Personality 1945, 14 3-23). The study was conducted from January 2024 to April 2024. The sample consisted of 42 male patients hospitalised with multiple cardiac pathology, whose average age was 49.40±7.71.

**Results:**

Patients with cardiovascular diseases mostly demonstrated middle level of the adherence to treatment (61.17±18.53%), twelve (30%) participants were defined as low-adherent, nine (22.5%) were high-adherent. The component of health anxiety known as vigilance to bodily sensations was found to be positively associated with the adherence to treatment (r=0.316, p=0.047). Conscientiousness was the only personality trait to demonstrate significant positive associations with the adherence (r=0.378, p=0.023). More interestingly, adherence to treatment appears to be positively associated with need-persistent and intropunitive frustration reactions (r=0.428, p=0.013; r=0.459, p=0.007) and negatively associated with extrapunitive frustration reactions (r=-0.409, p=0.004). Assessment of defense mechanisms reveals positive associations between overall defense maturity and adherence to treatment (r=0.388, p=0.021), indicating that low-adherent patients are more inclined to use less mature defenses.

**Conclusions:**

Thus, adherence to treatment in patients with cardiovascular diseases is associated with greater vigilance to bodily sensations, conscientiousness, defense maturity, use of need-persistent and intropunitive frustration reactions and lesser use of extrapunitive frustration reactions.

**Disclosure of Interest:**

None Declared

